# Investigation of canine extracellular vesicles in diffuse large B-cell lymphomas

**DOI:** 10.1371/journal.pone.0274261

**Published:** 2022-09-20

**Authors:** Marek Kulka, Kieran Brennan, Margaret Mc Gee

**Affiliations:** 1 Department of Pathology and Veterinary Diagnostics, Institute of Veterinary Medicine, Warsaw University of Life Sciences, Warsaw, Poland; 2 UCD School of Biomolecular and Biomedical Science, Conway Institute, University College Dublin, Dublin, Ireland; Texas Tech University Health Science, Lubbock, UNITED STATES

## Abstract

Diffuse large B-cell lymphomas (DLBCLs) are the most common lymphoproliferative diseases in dogs. DLBCL diagnosis to date has relied on histopathological analysis; however liquid biopsies have gained attention in recent years as a source of diagnostic and prognostic information. Liquid biopsies can be a source of circulating DNA, miRNA, circulating tumour cells or extracellular vesicles (EVs). In this study EVs were isolated from the plasma of healthy dogs, and dogs with lymphoma, and adenocarcinoma by iodixanol density gradient centrifugation. These EVs were positive for the EV markers CD63 and TSG101 and the pan-B cell markers CD79a, CD21, CD45, CD20. NTA analysis revealed that the DLBCL and adenocarcinoma dogs had elevated plasma EVs relative to the healthy dogs. Furthermore, the modal size of lymphoma EVs had decreased relative to healthy dogs while adenocarcinoma EVs were unchanged. This study demonstrates that the plasma EV population is altered in canine lymphoma patients in a manner similar to previous studies on human lymphomas. The similar changes to the EV population in dogs, together with the similar pathological features and treatment protocols in canine and human non-Hodgkin lymphomas would make dogs a good comparative model for studying the role of EVs in DLBCL development and progression.

## Introduction

Lymphoproliferative diseases are the most common neoplasmatic diseases in dogs [1]. The majority of canine lymphomas are diffuse large B-cell lymphomas (DLBCLs), characterized by a diffuse proliferation of large neoplastic lymphoid cells with a nuclear size more than twice the size of a red blood cell. There are two common types [2] the immunoblast and centroblastic variants, where the centroblastic variant is the most frequent and described as proliferation of large lymphoid cells similar to proliferating cells of the germinal center with oval to round vesicular nuclei (fine chromatin, multiple nucleoli) and small amount of basophilic cytoplasm. The immunoblastic variant [2], is more aggressive and described as round cells with centrally located nucleoli and a broad rim of basophilic cytoplasm [3].

Lymphomas initially present with enlargement of peripheral lymph nodes. When the internal lymph nodes are affected by the disease, then symptoms such as dyspnea, cough, and fatigue may occur. Further development of DLBCL is associated with organ infiltration (liver, spleen, bone marrow). Patients may show non-regenerative or regenerative anemias and lymphocytosis or lymphopenia [2, 4]. The ’gold standard’ treatment for canine lymphoma consists of a multi-agent, CHOP-based chemotherapy protocol (cyclophosphamide, doxorubicin, vincristine, and prednisone) [5, 6]. The similar pathological features and treatment protocols in canine and human non-Hodgkin lymphomas make dogs a good large comparative model in studying the DLBCLs [7]. Whereas the pathology of lymphomas is well recognized, the majority of current research is focused on early cancer diagnosis. Liquid biopsies are starting to play an important role in defining the first signs of lymphoproliferation. This minimally invasive approach can be used to monitor different components in blood such as circulating tumour DNA, miRNA and more recently extracellular vesicles (EVs) [8].

EVs are a heterogeneous group of nanometer-sized, membrane-bounded vesicles that play an important role in intercellular communications [9]. They originate from the inward budding of multivesicular endosomes (40–150 nm exosomes) or outward budding from the plasma membrane (>100 nm microvesicles or apoptotic bodies) [10, 11]. EVs are secreted by presumably all cell types and are found in most biofluids, including blood, serum, urine, and cerebral spinal fluid [12, 13]. EVs can transfer a wide range of nucleic acids, lipids, and soluble and membrane-associated proteins, and have been shown to play a pivotal role in normal physiology and disease, including maintenance of cellular homeostasis, regulation of gene transcription, activation and modulation of immune response and cancer progression [12–14].

Tumour-derived EVs have been reported to enhance cell proliferation, invasion, migration, and angiogenesis [15] and can influence drug resistance by transferring multidrug-resistant proteins, miRNAs, and exporting chemotherapeutic drugs [16, 17]. Several studies have examined exosome-derived RNA as a potential biomarker in DLBCL [18–25]. DLBCL-derived EVs can regulate several functions of natural killer cells [25] as well as macrophage polarization [26].

Currently, DLBCL diagnosis relies on tissue specimen examination, which is invasive and expensive. EV encapsulation can improve protein and miRNA stability in biofluids making EVs an attractive source of biomarkers for the development of non-invasive tests for the early diagnosis or follow-up and the prediction of treatment response [27–29]. Liquid biopsies could improve lymphoma management due to the non-invasive nature, and the ability to reflect spatial inter- and intra-tumour heterogeneity, and the possibility of repeated measurements through longitudinal profiling without the need for a tissue biopsy.

The aim of this preliminary study is to examine changes in the EV population in patients with DLBCL and how these changes in the EV population can be applied in the diagnosis of DLBCL.

## Materials and methods

### Animals, material collection

Blood was collected by certified veterinary surgeons during medical checkups or before planned surgery after obtaining verbal consent from the owner. Each patient was clinically evaluated. One group consisted of 14 healthy dogs with an age ranging from 1 year and 2 months to 7 years 3 months old. A second group consisted of 13 patients with an age ranging from 4 years to 15 years and 4 months that were diagnosed with diffuse large B-cell lymphoma (DLBCL) based on the lymph nodes fine needle biopsy results (cytological analysis and 12 patients by immunohistochemistry and 1 with PARR). During clinical examination patients presented with lymphadenopathy, 8 had cough and dyspnea. The third group consisted of two patients aged 7–8 years that were diagnosed with adenocarcinoma, which presented with mammary tumours in one of their milk strips.

Blood samples were taken from the cephalic vein and collected into appropriate collection test tubes with EDTA-K2 and into clotting test tubes. Serum was aspirated after centrifugation of clotted samples. The whole blood was tested for CBC and serum for basic biochemical panels: alanine aminotransferase (ALT), aspartate aminotransferase (AST), alkaline phosphatase (AP), urea, creatinine, total protein (TP), albumins, globulins, glucose, lipase also blood smears were prepared stained with May-Grunwald Giemsa stain (according to the manufacturer’s recommendations) and assessed. The plasma samples were separated from blood cells using Lymphoprep^™^ (Stem Cell Technologies). The plasma fraction from the Lymphoprep^™^ was centrifuged at 600×g at 4°C, for 10 min and then the supernatant was centrifuged at 2,000×g at 4°C, for 20 min. The platelet free plasma was stored at -80°C for the EV isolations.

### Ultracentrifugation

All ultracentrifugations were performed using a Beckman Coulter Optima MAX-UP ultracentrifuge (stopping without break), with centrifugation durations based on a “50 nm cut-off size”, as described in Livshits et al., with an additional 5 min added to allow the rotor to come up to speed. Plasma samples were defrosted and transferred to a 13.5 ml Beckman Coulter ultracentrifuge tube (Prod. No. 355630) [30]. The plasma was diluted to 8.6 ml with particle-free PBS for ultracentrifugation. The tubes were centrifuged at 120000 g (RCF avg, 40800 rpm) for 2 hours at 20°C, using a Beckman Coulter MLA-55 rotor. The supernatant was removed, and the EV pellet was resuspended in 100 μl residual PBS.

### Iodixanol density gradient centrifugation

Density gradient centrifugation was performed using a modified protocol from [31, 32]. A 54% iodixanol-PBS working solution was prepared by diluting a stock solution of OptiPrep^™^ (60% (w/v) aqueous iodixanol from Axis-Shield PoC, Norway) with 10x particle-free PBS (Gibco, Waltham, MA, USA). Iodixanol solutions (41% (w/v), 35% (w/v)), were prepared by diluting the 54% iodixanol-PBS working solution in 1x particle-free PBS (Gibco, Waltham, MA, USA). To form the gradient, firstly a homogenous 41% (w/v) base layer of the gradient (estimated density ~1.223 g/ml) was produced by adding 336 μl of the 54% iodixanol-PBS working solution to a 16 × 76 mm thinwall, ultra-Clear^™^ tube (Beckman Coulter), together with resuspended pellet. Next, 1.7 ml 35% (w/v) iodixanol (estimated density ~1.192 g/ml), and 1.7 ml 14% iodixanol (estimated density ~1.08 g/ml) were layered successively on top of the vesicle suspension. Centrifugation was performed at 219,373g (RCF avg, 55000 rpm) for 16 h at 20°C in Beckman Coulter Optima MAX-UP ultracentrifuge, using Beckman Coulter MLA-55 rotor (stopping without break). Fractions (~200 μl) were collected from the top of the tube and then 50 μl of each fraction was pipetted into a 96 well plate and the absorbance of the fractions was measured at 340 against an iodixanol standard curve to determine the fraction density. The fractions with densities between 1.08–1.19 g/ml were combined and diluted to a density <1.03 g/ml with particle-free PBS and the diluted fractions were centrifuged at 120000 g (RCF avg, 40800 rpm using Beckman Coulter MLA-55 rotor) for 2 hours at 4°C in Beckman Coulter Optima MAX-UP ultracentrifuge (stopping without break). The supernatant was removed, and the EV pellets were resuspended in 100 μl residual PBS.

### NTA analysis

Particle number and size distribution in plasma samples was determined by nanoparticle tracking analysis (NTA) using a NanoSight LM10 (Malvern Panalytical, UK) configured with a 405 nm laser and a high sensitivity scientific CMOS camera. Samples were diluted (plasma 20:400) in particle-free PBS (Gibco, Waltham, MA, USA). Samples were analyzed at 24°C and 15×60s videos were captured with a camera level of 15. Data was analyzed using NTA 3.2 Dev Build 3.2.16 software with a detection threshold of 5.

### Western blot analysis

15 μg protein isolated from the serum EV fraction was separated by SDS–PAGE (10% polyacrylamide) according to a standard protocol. The polypeptides were electroblotted onto nitrocellulose membrane (Immobilon^®^-FL PVDF membrane) and probed with a primary antibody overnight at 4° C. Alix (1:200; Santa Cruz sc-53538), Tsg101 (1:500; Abcam ab83), CD63 (1:5000; ABIN 1440014), CD79a (1:500; ABIN 2472431), CD20 (1:2000; ABIN 2717455), CD21 (1:500; ABIN 94032), CD45 (1 μg/ml; ABIN 6940460) antibodies were used as primary antibodies. Anti-goat (for CD63) or anti-mouse (for the rest) antibodies conjugated to horseradish peroxidase (Bio-Rad, Hercules, CA, USA) were used as secondary antibodies at a dilution of 1/5000 or 1/10000, respectively. Visualization was performed with a chemiluminescent reagent system (Clarity^™^Western ECL, Bio-Rad, Hercules, CA, USA). Blots were quantified by densitometry using Quantity One 4.6.2 software (Bio-Rad Ltd.).

### Statistical analysis

All groups were checked for normal distribution and for comparisons an unpaired T-test was used. *P* values were considered significant when < 0.05.

## Results

All blood morphological and biochemical parameters were within reference values [33] with no significant difference between healthy control dogs and dogs with adenocarcinoma. Within the DLBCL group 2 patients displayed normocytic normochromic non regenerative anemia, and 3 displayed mild lymphopenia. Biochemical parameters were within normal limits, with the one exception where two dogs displayed a mild increase in ALT.

**Fig 1:**
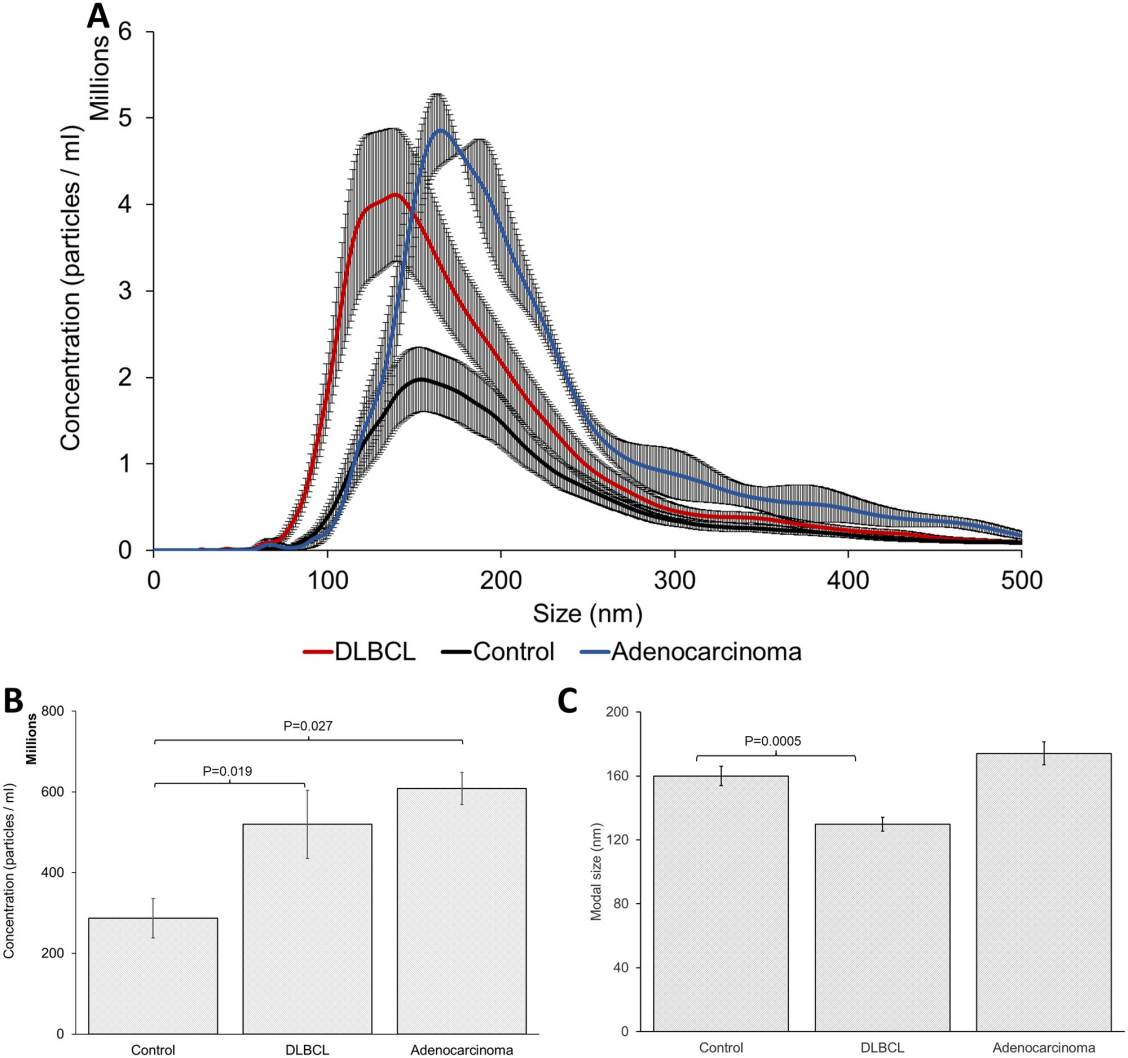
NanoSight analysis of particle size distribution of EVs isolated from canine plasma. (A) Line graph of healthy control, DLBCL and adenocarcinoma patient particle size distribution. (B) Bar graph of total EV concentration of particles / ml and (C) modal size of healthy control, DLBCL and adenocarcinoma patient EV samples.

Plasma was prepared by depletion of cells and platelets by centrifugation. Plasma EVs were isolated by ultracentrifugation and iodixanol density gradient ultracentrifugation. The EVs were resuspended in PBS and particle size and size distribution of the canine EVs were examined by NTA analysis (Fig 1A). DLBCL patients had higher EV concentrations than healthy controls, with DLBCL patient plasma having 5.19 ± 3 x 10^8^ particles / ml and healthy control plasma having 2.86 ± 1.8 x 10^8^ particles / ml (*P* = 0.019) (Fig 1B). DLBCLs EVs also had a smaller modal size (129.7 ± 15.6 nm) when compared to healthy controls (159.9 ± 22.6 nm) (*P* = 0.0013) (Fig 1C). Adenocarcinoma patients had higher EV concentration (6.08 ± 5.6 x 10^8^ particles / ml) than healthy controls (*P* = 0.027), however, no change in modal size was observed.

**Fig 2:**
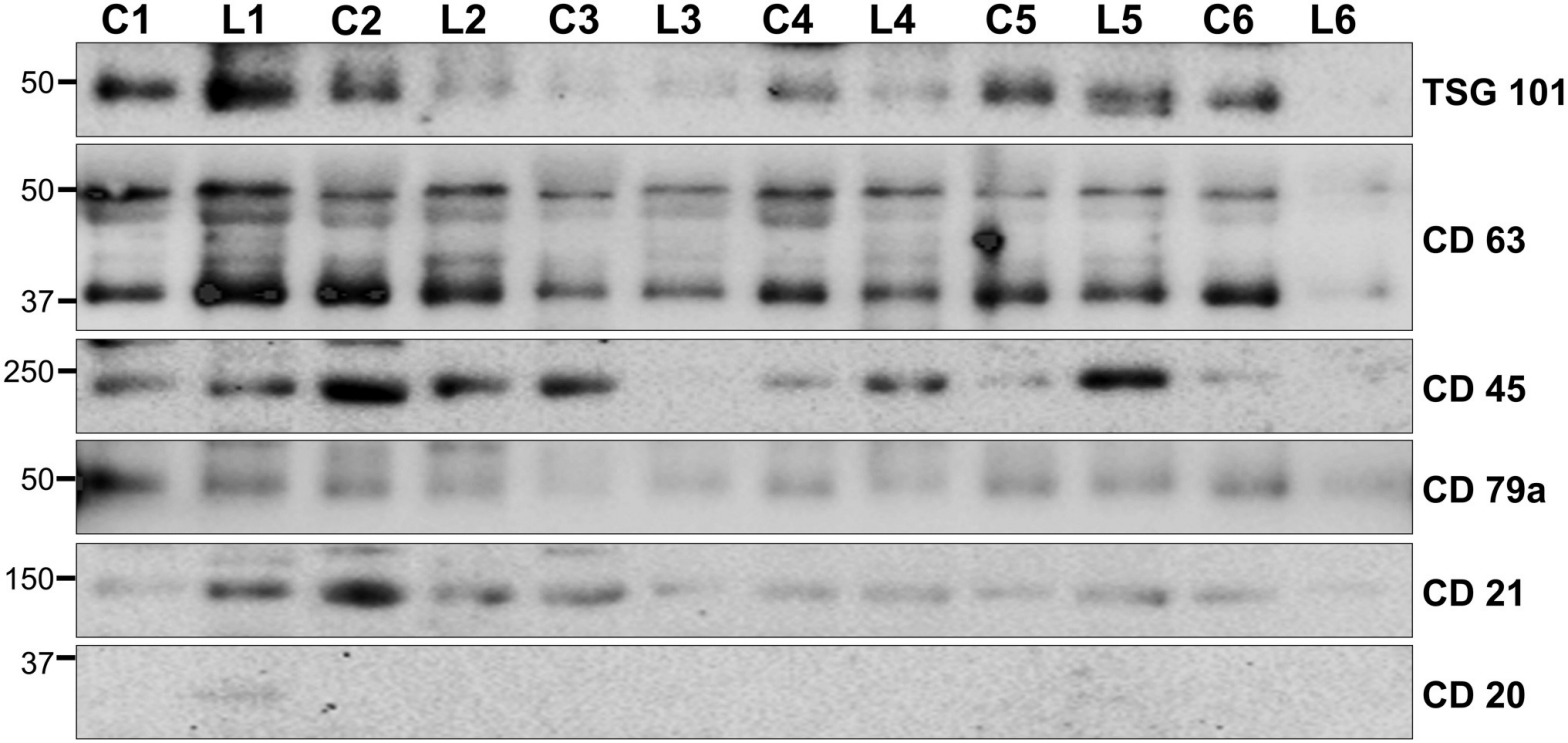
Western blot analysis of protein lysates from isolated from canine plasma.

Western blot analysis was performed using an equal amount of protein from each serum EV sample. The EVs markers; Tsg101 and CD63, and the lymphoma markers; CD45, CD79a, CD21, were detected in all EV samples. However, only one DLBCL patient was positive for CD20 in EVs (Fig 2).

15 μg protein from6 lymphoma (L1-6) and 6 control (C1-6) serum EV samples were analyzed alongside a molecular weight marker (Bio-Rad # 161–0375) by SDS–PAGE and western blot analysis. The membranes were probed with anti-TSG101 (49 kDa, 1:500; Abcam ab83), anti-CD63 (40–60 kDa, 1:5000; ABIN 1440014), anti-CD79a (44–50 kDa, 1:500; ABIN 2472431), anti-CD20 (30–50 kDa, 1:2000; ABIN 2717455), anti-CD21 (145 kDa, ABIN 94032), anti-CD45 (180–220 kDa, 1 μg/ml; ABIN 6940460). The EVs markers TSG 101, CD63 and lymphoma markers CD45, CD79a, CD21 were detected in patients with DLBCLs (L) and healthy dogs (C), CD20 was detected in one patient with DLBCL.

## Discussion

EVs play a role in cell-to-cell communication, promotion of metastasis and immune suppression. EVs have gained a lot of interest as a source of diagnostic and prognostic biomarkers for human cancer research and are becoming more important in veterinary medicine. EVs are a promising option for lymphoma monitoring as the EV content can reflect the cell from which they originate and they are readily accessible in biofluids without the need for tissue biopsy. Canine DLBCL patients were found to have an elevated concentration of serum EVs relative to healthy patients. A similar observation has been made in human hematological neoplastic disorders, with elevated plasma EV levels observed in multiple myeloma, Hodgkin’s lymphoma and to a lesser degree in non-Hodgkin’s lymphoma and chronic lymphocytic leukemia [34]. A higher concentration of EVs was also observed in dogs with adenocarcinoma, which confirmed studies made on canine and feline mammary tumour cell lines [35]. In addition to increased EV concentration, DLBCL patients had a smaller EV modal size relative to healthy dogs, which was comparable to the decreased EV modal size observed in human hematological cancers [34]. It is not possible to distinguish whether this increase in smaller EVs is due to an upregulation of the exosome biogenesis pathways or whether it is due to increased small microvesicle budding from the plasma membrane [36] as specific markers to distinguish these two pathways do not currently exist. All samples were positive for the EV membrane marker CD63 and the intra-EV marker TSG101 [37]. DLBCL has no specific immunophenotype for either canine or human cancers; however, they express pan-B markers including CD79a, CD21, CD45, CD20 [1]. In this study, the DLBCL patient EVs were positive for CD79a, CD21, and CD45, however, only one EV sample was positive for CD20, suggesting some diversity within the DLBCL group. This heterogeneity in B-cell expression profile may help diagnostic approaches for DLBCL patients in the future however, a larger study would be needed to assess the diagnostic significance of CD20 in DLBCL plasma EVs. The presence of these lymphoma markers in control patients indicates that EVs from normal immune cells are also present in the plasma EV population. Furthermore, the lack of a strong correlation between the NTA data and the western blot results suggests that alternative protein markers will need to be examined to determine if it is possible to identify the population of EVs that is increasing in lymphoma patients.

In conclusion, we have found that canine DLBCL patients have elevated levels of plasma EVs, that express lymphocyte markers. These EVs had a smaller modal size relative to healthy controls suggesting that not all EV sizes were increased, but instead the production of small EVs was upregulated. This observation is similar to previous studies on human lymphomas [7, 8, 18]. The fact that canine and human DLBCL patients share similar pathological features and treatment protocols suggests they may also share the same EV signaling pathways, making dogs a good large comparative model to study DLBCL.

## Supporting information

S1 File Supplemental western blot figure. (PPTX)

S2 File Supplementary NTA figure. (XLS)
