# Supplementary material for: Investigation of canine extracellular vesicles in diffuse large B-cell lymphomas
Source: PLoS One. 2022 Sep 20;17(9):e0274261. doi: 10.1371/journal.pone.0274261 (PMC9488776; doi:10.1371/journal.pone.0274261)
Supplement: S1 File — (PPTX) [file pone.0274261.s001.pptx]

## Slide 1
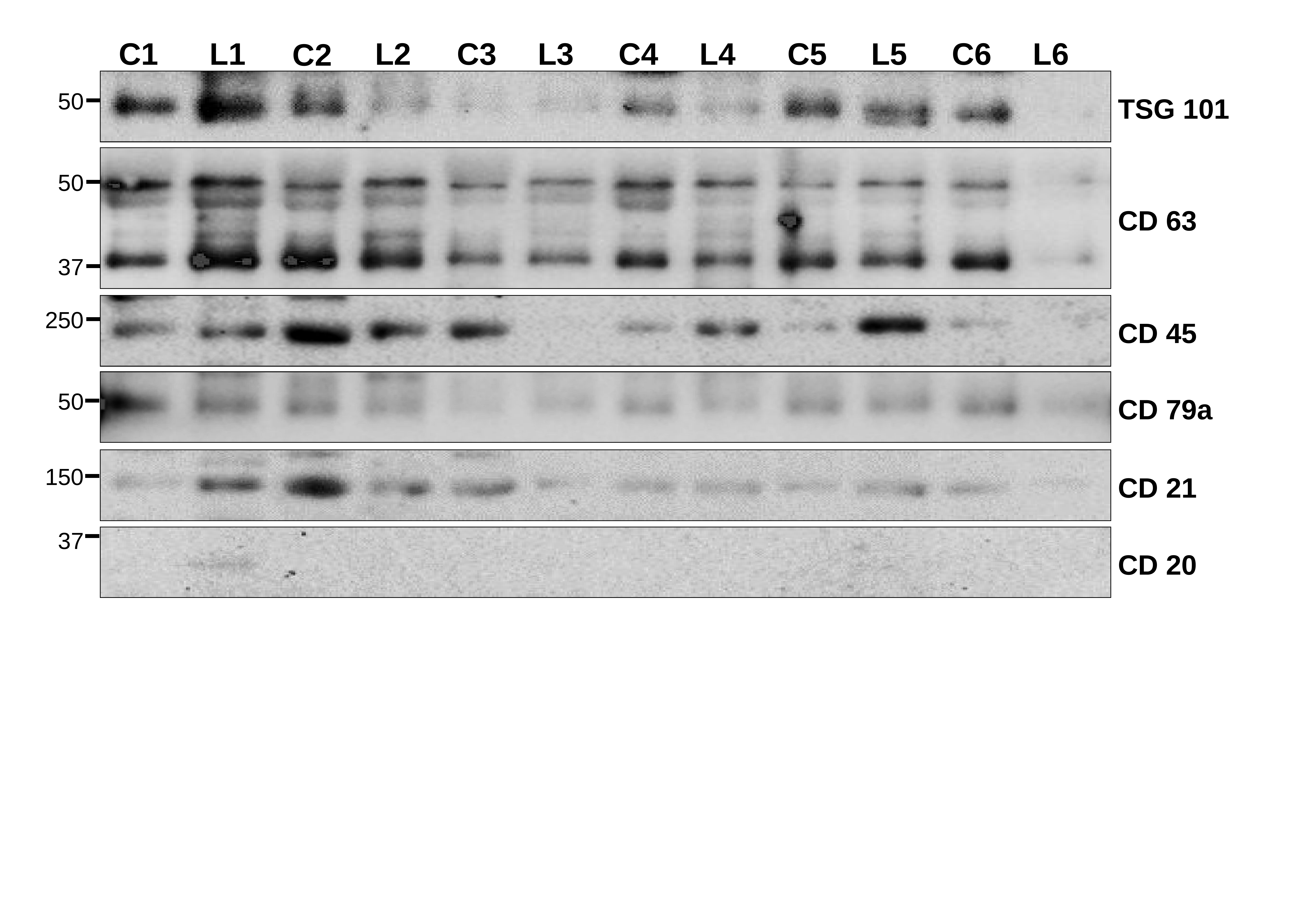

C1
L1
L2
C3
L3
C4
L4
C5
L5
C6
L6
C2
50
TSG 101
50
CD 63
37
250
CD 45
50
CD 79a
150
CD 21
37
CD 20

## Slide 2
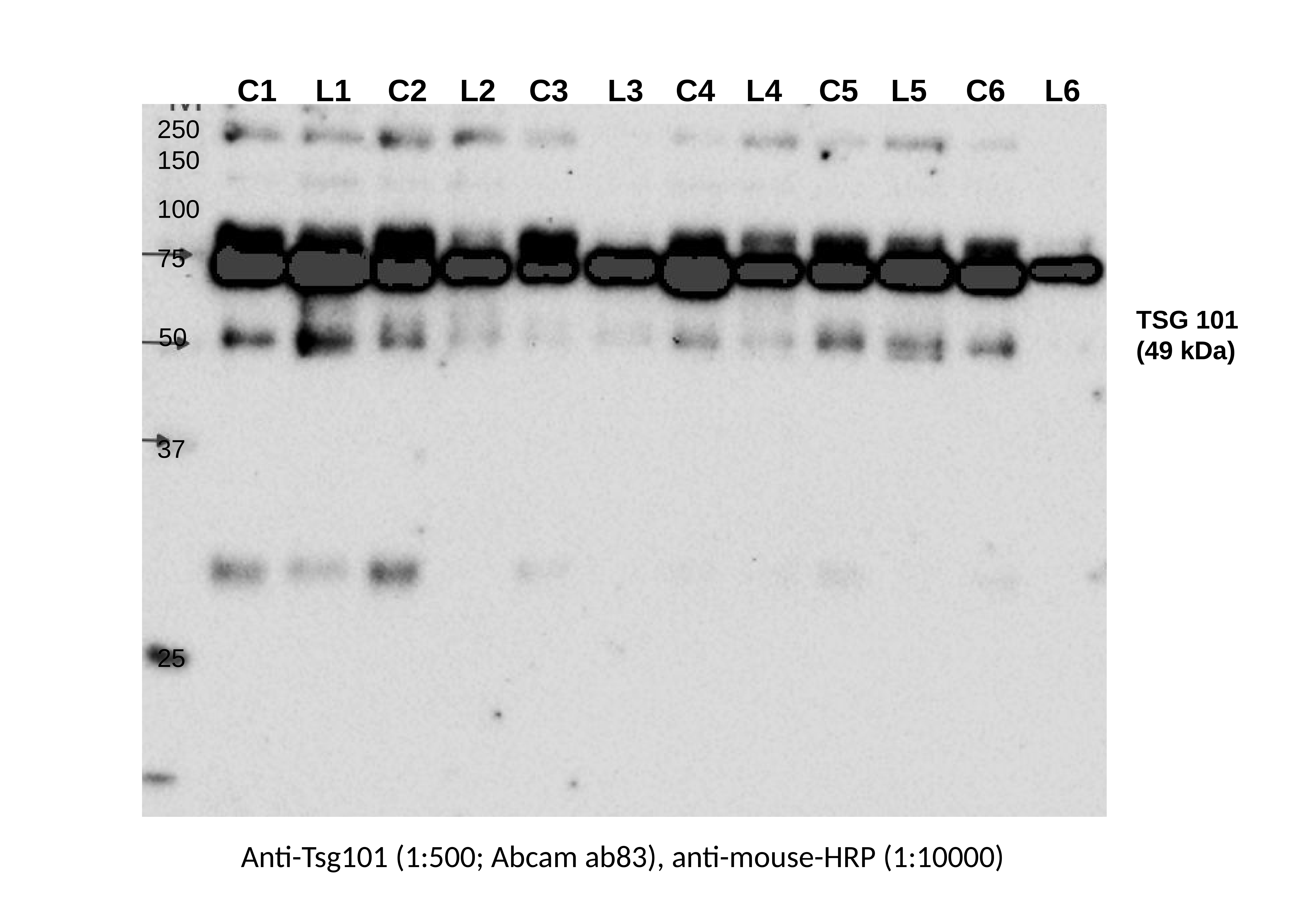

C1
L1
C2
L2
C3
L3
C4
L4
C5
L5
C6
L6
250
150
100
75
TSG 101
(49 kDa)
50
37
25
Anti-Tsg101 (1:500; Abcam ab83), anti-mouse-HRP (1:10000)

## Slide 3
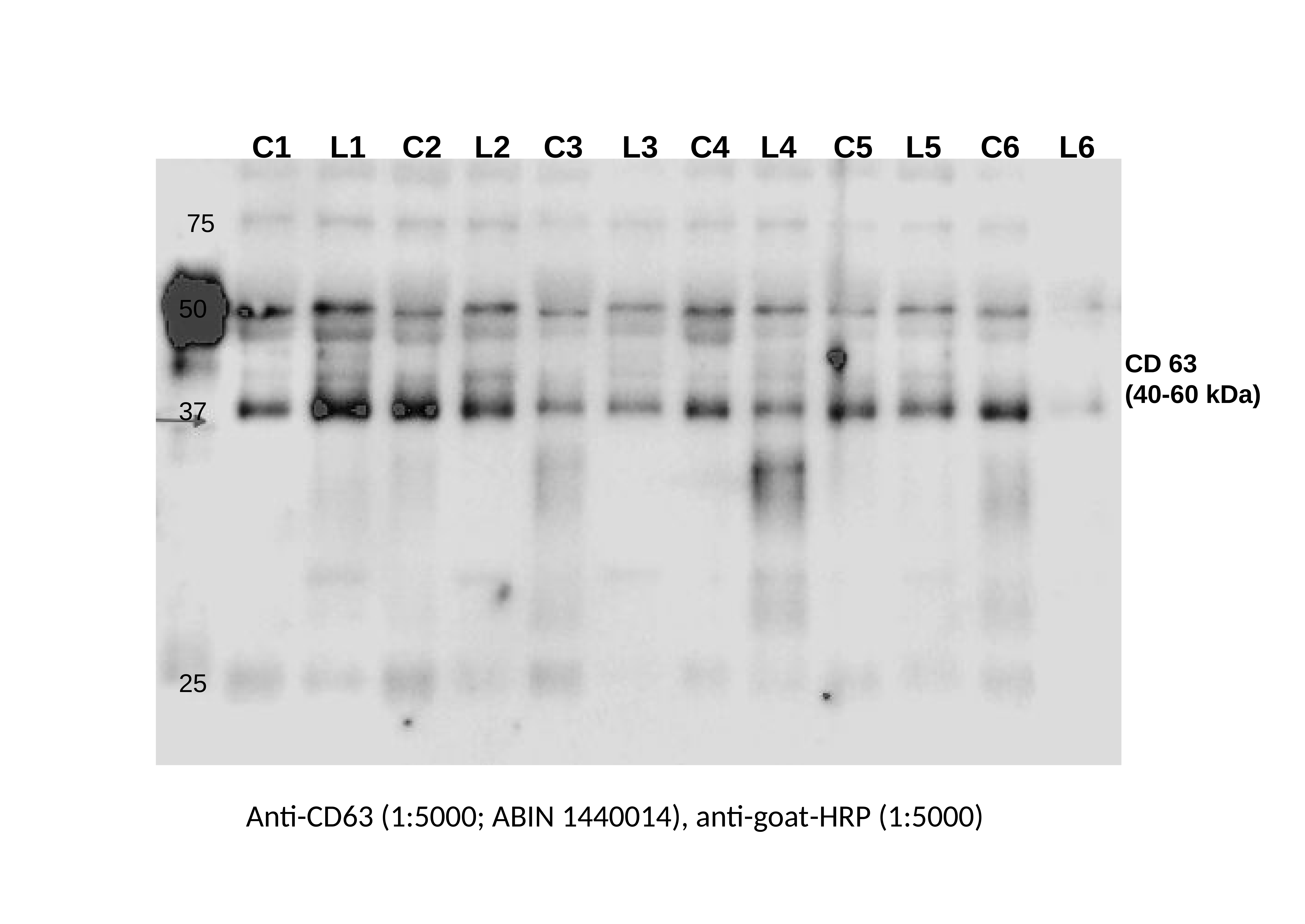

C1
L1
C2
L2
C3
L3
C4
L4
C5
L5
C6
L6
75
50
CD 63
(40-60 kDa)
37
25
Anti-CD63 (1:5000; ABIN 1440014), anti-goat-HRP (1:5000)

## Slide 4
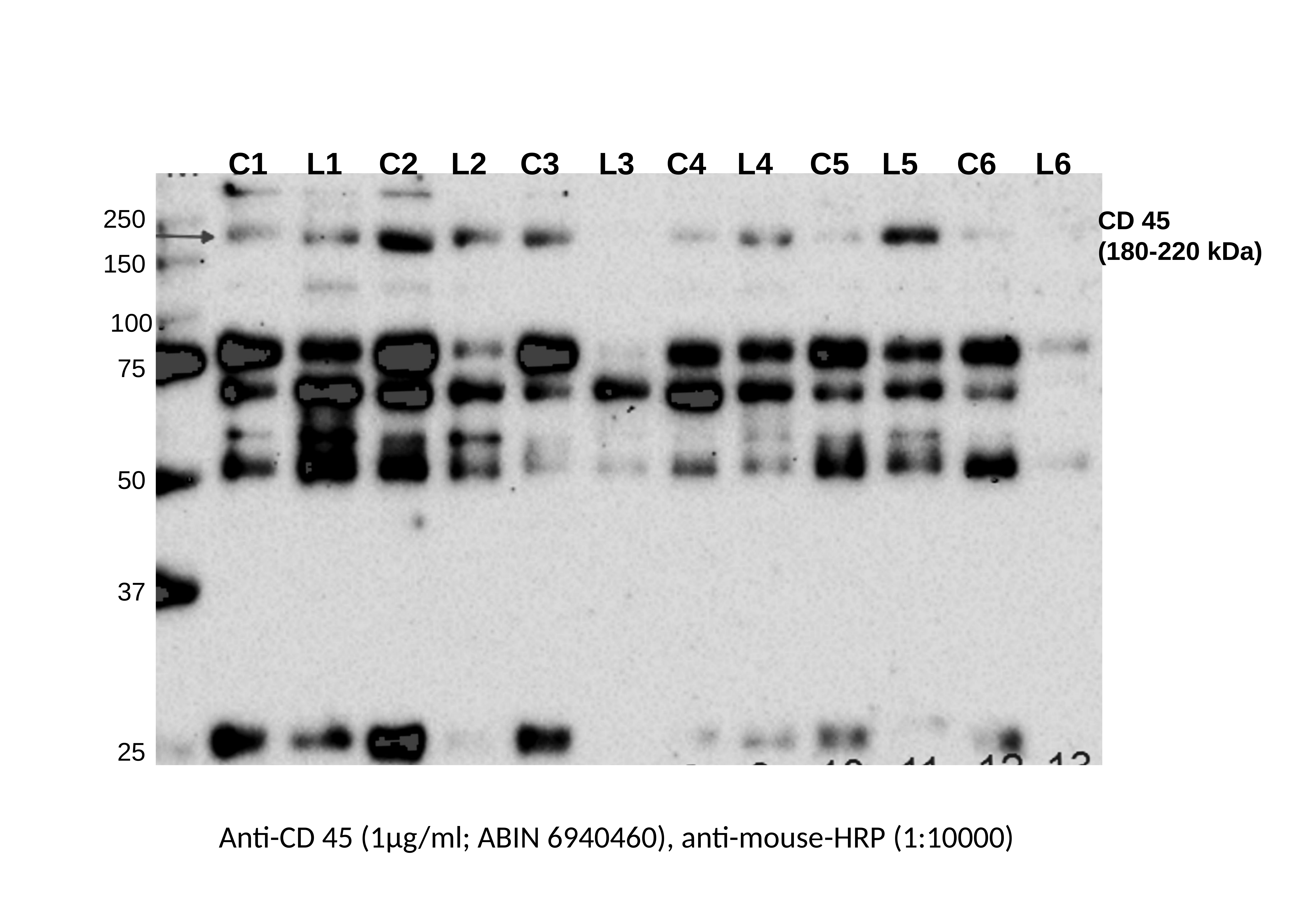

C1
L1
C2
L2
C3
L3
C4
L4
C5
L5
C6
L6
250
CD 45
(180-220 kDa)
150
100
75
50
37
25
Anti-CD 45 (1μg/ml; ABIN 6940460), anti-mouse-HRP (1:10000)

## Slide 5
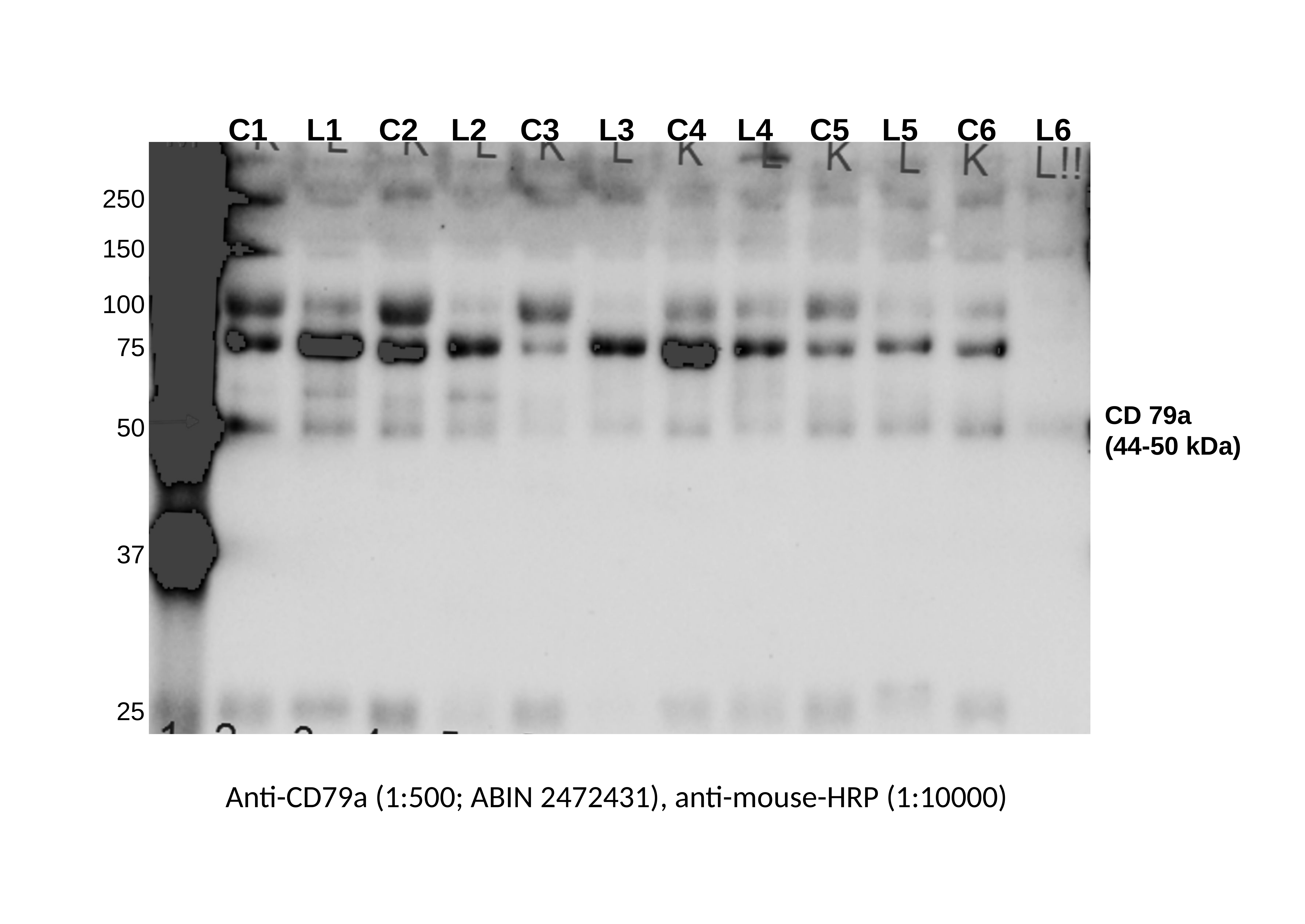

C1
L1
C2
L2
C3
L3
C4
L4
C5
L5
C6
L6
250
150
100
75
CD 79a
(44-50 kDa)
50
37
25
Anti-CD79a (1:500; ABIN 2472431), anti-mouse-HRP (1:10000)

## Slide 6
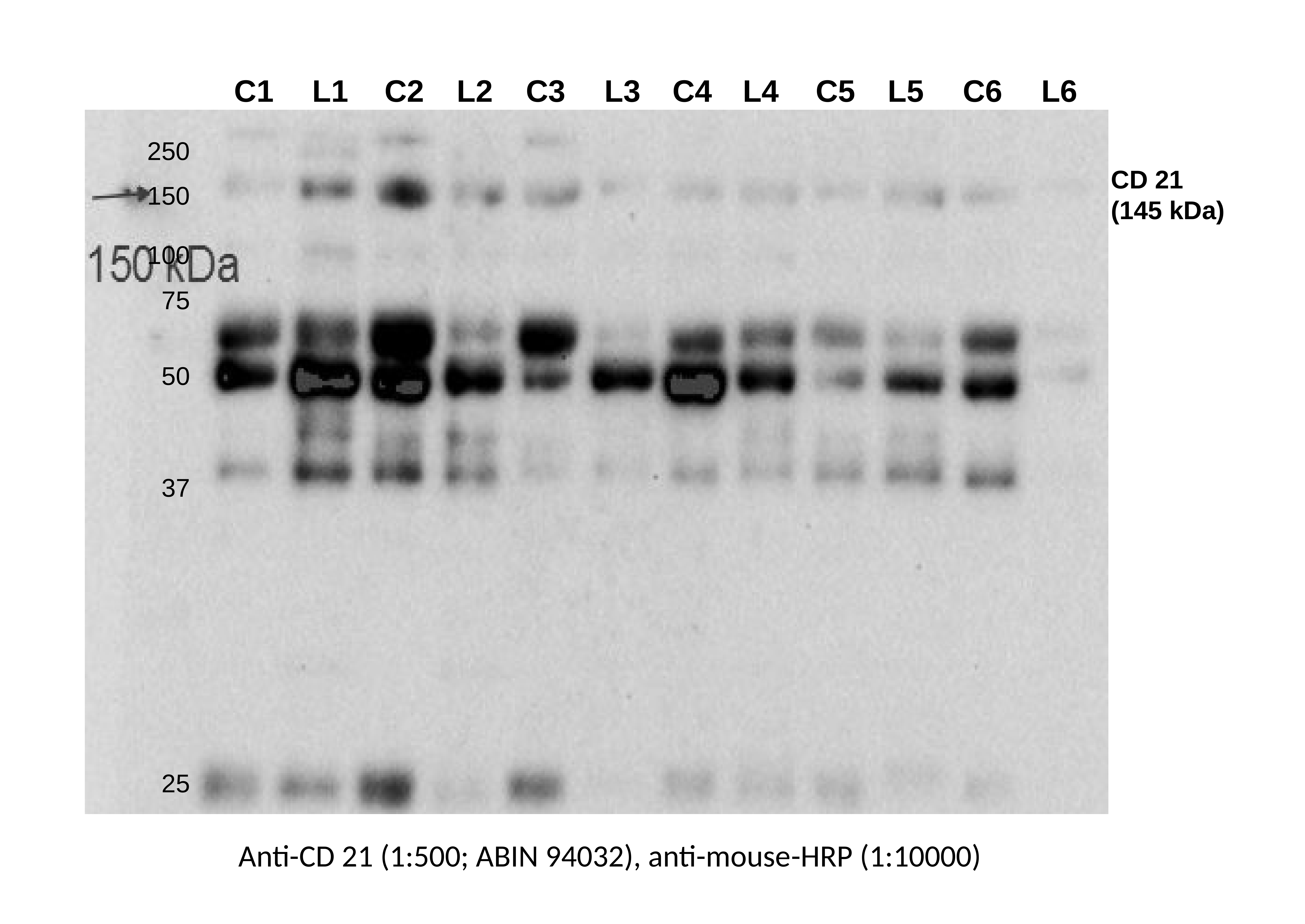

C1
L1
C2
L2
C3
L3
C4
L4
C5
L5
C6
L6
250
CD 21
(145 kDa)
150
100
75
50
37
25
Anti-CD 21 (1:500; ABIN 94032), anti-mouse-HRP (1:10000)

## Slide 7
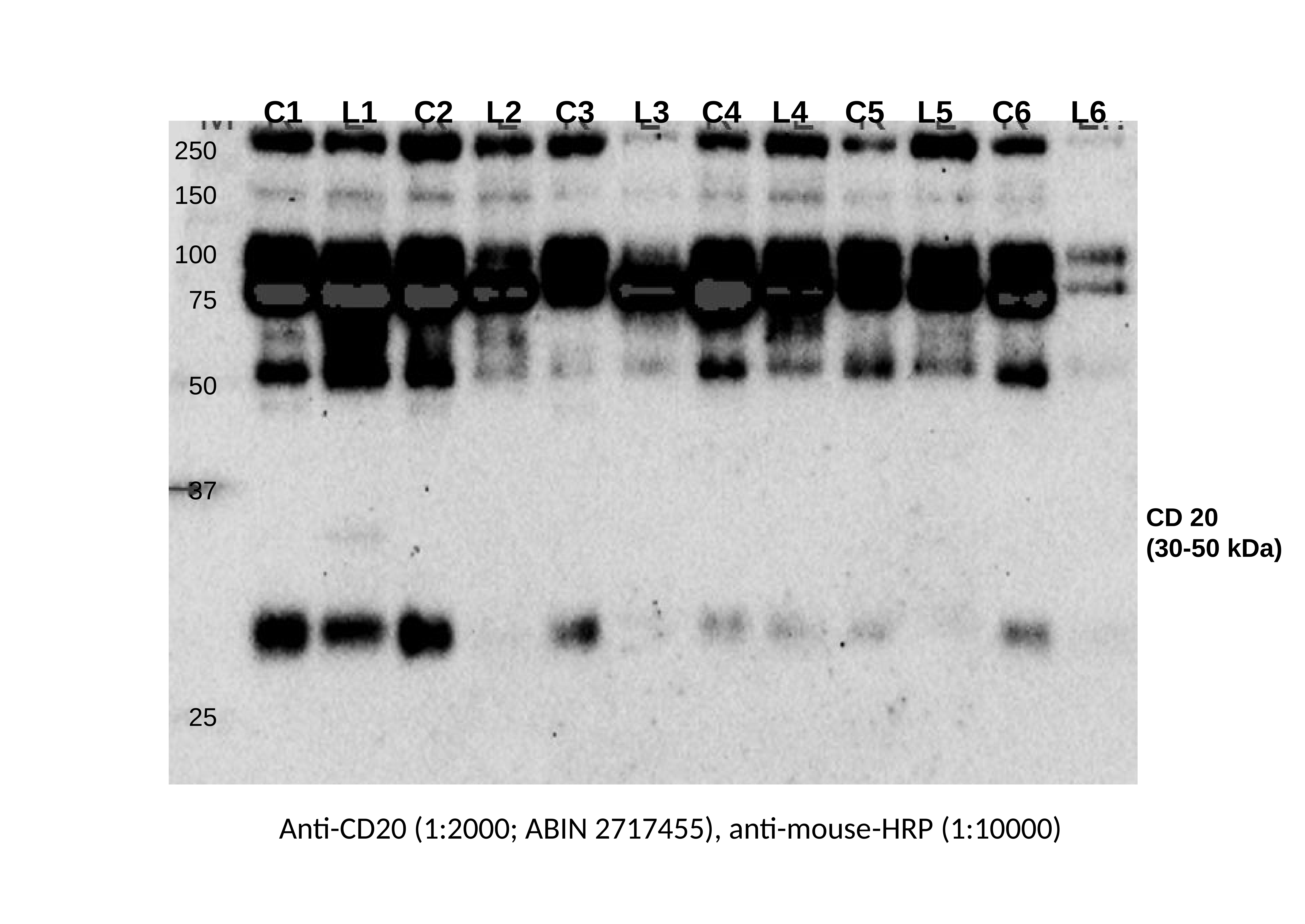

C1
L1
C2
L2
C3
L3
C4
L4
C5
L5
C6
L6
250
150
100
75
50
37
CD 20
(30-50 kDa)
25
Anti-CD20 (1:2000; ABIN 2717455), anti-mouse-HRP (1:10000)
